# Identification of an Immunogenic Broadly Inhibitory Surface Epitope of the Plasmodium vivax Duffy Binding Protein Ligand Domain

**DOI:** 10.1128/mSphere.00194-19

**Published:** 2019-05-15

**Authors:** Miriam T. George, Jesse L. Schloegel, Francis B. Ntumngia, Samantha J. Barnes, Christopher L. King, Joanne L. Casey, Michael Foley, John H. Adams

**Affiliations:** a Center for Global Health and Infectious Disease Research, Department of Global Health, University of South Florida, Tampa, Florida, USA; b Center for Global Health and Diseases, Case Western Reserve University, Cleveland, Ohio, USA; c Department of Biochemistry, La Trobe University, Melbourne, Victoria, Australia; Johns Hopkins Bloomberg School of Public Health

**Keywords:** DBPII, *Plasmodium vivax*, epitope mapping, malaria, vaccine

## Abstract

The Plasmodium vivax Duffy binding protein region II (DBPII) is a vital ligand for the parasite’s invasion of reticulocytes, thereby making this molecule an attractive vaccine candidate against vivax malaria. However, strain-specific immunity due to DBPII allelic variation in Bc epitopes may complicate vaccine efficacy, suggesting that an effective DBPII vaccine needs to target conserved epitopes that are potential targets of strain-transcending neutralizing immunity. The minimal epitopes reactive with functionally inhibitory anti-DBPII monoclonal antibody (MAb) 3C9 and noninhibitory anti-DBPII MAb 3D10 were mapped using phage display expression libraries, since previous attempts to deduce the 3C9 epitope by cocrystallographic methods failed. Inhibitory MAb 3C9 binds to a conserved conformation-dependent epitope in subdomain 3, while noninhibitory MAb 3D10 binds to a linear epitope in subdomain 1 of DBPII, consistent with previous studies. Immunogenicity studies using synthetic linear peptides of the minimal epitopes determined that the 3C9 epitope, but not the 3D10 epitope, could induce functionally inhibitory anti-DBPII antibodies. Therefore, the highly conserved binding-inhibitory 3C9 epitope offers the potential as a component in a broadly inhibitory, strain-transcending DBP subunit vaccine.

**IMPORTANCE** Vivax malaria is the second leading cause of malaria worldwide and the major cause of non-African malaria. Unfortunately, efforts to develop antimalarial vaccines specifically targeting Plasmodium vivax have been largely neglected, and few candidates have progressed into clinical trials. The Duffy binding protein is considered a leading blood-stage vaccine candidate because this ligand’s recognition of the Duffy blood group reticulocyte surface receptor is considered essential for infection. This study identifies a new target epitope on the ligand’s surface that may serve as the target of vaccine-induced binding-inhibitory antibody (BIAb). Understanding the potential targets of vaccine protection will be important for development of an effective vaccine.

## Abstract (precis)

Vivax malaria is the second leading cause of malaria worldwide and the major cause of non-African malaria. Unfortunately, efforts to develop antimalarial vaccines specifically targeting Plasmodium vivax have been largely neglected, and few candidates have progressed into clinical trials. The Duffy binding protein is considered a leading blood-stage vaccine candidate because this ligand’s recognition of the Duffy blood group reticulocyte surface receptor is considered essential for infection. This study identifies a new target epitope on the ligand’s surface that may serve as the target of vaccine-induced binding-inhibitory antibody (BIAb). Understanding the potential targets of vaccine protection will be important for development of an effective vaccine.

## INTRODUCTION

Plasmodium vivax is the second most prevalent cause of human malaria and is the most widely distributed, placing about 40% of the world’s population at risk (1). Although vivax malaria has historically been called “benign tertian malaria” (2), there have been increasing reports of clinical severity with emerging virulent forms of the parasite (3, 4), recurrent clinical episodes due to reactivation of the dormant forms in the liver (5), and widespread drug resistance (6–8), which potentially includes strains with low sensitivity to primaquine, the only drug against relapse, or in people with poor ability to metabolize the drug to its active form (9, 10). Therefore, there is an urgent need to develop new therapies, especially a vaccine to control and prevent vivax malaria.

Immunity to asexual blood-stage antigens plays an important role in controlling P. vivax infections, especially targets important for blocking blood-stage replication or reticulocyte reinfection. Merozoite antigens are believed to be an important component of naturally acquired immunity to blood-stage vivax malaria (11, 12), and they represent ideal candidates for vaccine-mediated immunity against blood-stage infection (13). The P. vivax Duffy binding protein (DBP) binds its cognate receptor, the Duffy antigen receptor for chemokines (DARC), on reticulocytes to form an irreversible junction critical for merozoite invasion of reticulocytes (14–19). Even though there have been a few P. vivax cases reported recently among Duffy-negative individuals (20–22), this phenomenon remains relatively restricted in prevalence. A gene duplication event, creating a paralog of the DBP ligand lost in some primate-adapted parasite strains (21, 23), seems to be an alternate invasion pathway that is secondary to or less efficient than DBP (24). Hence, DBPII is a prime candidate for vaccine-induced immunity against blood-stage P. vivax infection.

Plasmodium vivax DBP is a type I membrane protein localized to the merozoite’s micronemes and is a member of the Duffy binding-like erythrocyte binding protein (DBL-EBP) family (18). The DBL-EBPs are micronemal proteins that have similar sequences in their functional domains, including an N-terminal cysteine-rich ligand domain referred to as the Duffy binding-like domain (DBL) (17, 18, 25–28). For DBP, this principal determinant for receptor recognition is a 330-amino-acid also known as DBP region II (DBPII) (18, 29). Interestingly, crystal structural studies of DBPII, as well as the ligand domains of its Plasmodium falciparum homolog PfEBA175 (P. falciparum EBA175), revealed that the DBL domains form heterodimers as part of the receptor recognition process (26–28). It is the central region of DBPII important for dimerization that is also the most polymorphic region of DBP (30–32), occurring in a pattern consistent with high immune selection (32–35). Also, similar to other microbial ligands, the functionally important residues in the central region critical for erythrocyte binding are flanked by polymorphic residues not important for binding to the receptor (27, 35, 36). Therefore, the polymorphic nature of the central region of DBPII represents a potential challenge to developing a vaccine, since this variation contributes to strain specificity in naturally acquired immunity (35, 37–39).

In regions where malaria is endemic, individuals develop anti-DBPII antibodies, which increase with age, suggesting a boosting effect from repeated exposure to infection (40, 41). However, anti-DBPII antibodies tend to be weak, short-lived, and strain specific (41–43) with allelic differences appearing to be driven by immune selection (35, 38). Nonetheless, a few individuals do develop high titers of broadly inhibitory, strain-transcending anti-DBPII antibodies (30, 39, 41). These data are all consistent with the hypothesis that variation is an immune evasion mechanism responsible for strain-specific immunity and that stable, broadly inhibitory immunity is achieved when functionally inhibitory antibodies target conserved DBPII epitopes.

To map the immunoreactive surface of DBPII, we generated and characterized a panel of anti-DBPII murine monoclonal antibodies (MAbs) with different anti-DBPII titers and levels of inhibition (44). Using the most and least inhibitory anti-DBPII MAbs of this panel, we sought to identify a potential target of broadly binding-inhibitory antibody (BIAb). An important advance was achieved when MAb epitope cocrystal structures were solved for several of the highly inhibitory MAbs (45). Unfortunately, a high resolution of the epitope structure could not be elucidated for MAb 3C9, which is one of the most inhibitory MAbs.

In this study, we mapped the minimal reactive epitopes of anti-DBPII MAb 3C9 by screening DBPII gene fragment libraries expressed on M13 phage surface for minimal reactive peptide fragments. Phage display has been a useful tool for epitope mapping with the advantages of rapidly producing refolded protein, fused to phage coat, forming a traceable link back to the genotype (46, 47), and has been used to map epitopes on P. falciparum apical membrane antigen 1 (AMA1) (48–51), merozoite surface protein (MSP) (52), and circumsporozoite protein (CSP) (53–55). Additional immunogenicity studies demonstrated the ability of the 3C9 epitope, but not the 3D10 epitope, to elicit functionally inhibitory anti-DBPII serum antibodies that blocked DBPII-erythrocyte binding. The information derived from this study contributes to our understanding of a potential target for broadly inhibitory vaccine-elicited protective immunity that should be retained in the next generation of potent vaccines against asexual-stage vivax malaria. In particular, this study defines a potential immunogenic epitope of a DBPII vaccine that may be successful in boosting antibody responses targeted against conserved protective epitopes, with functional inhibition against broader allelic variants and diverse P. vivax strains.

## RESULTS

### Creation of DBPII gene random fragment phage displayed library.

**FIG 1:**
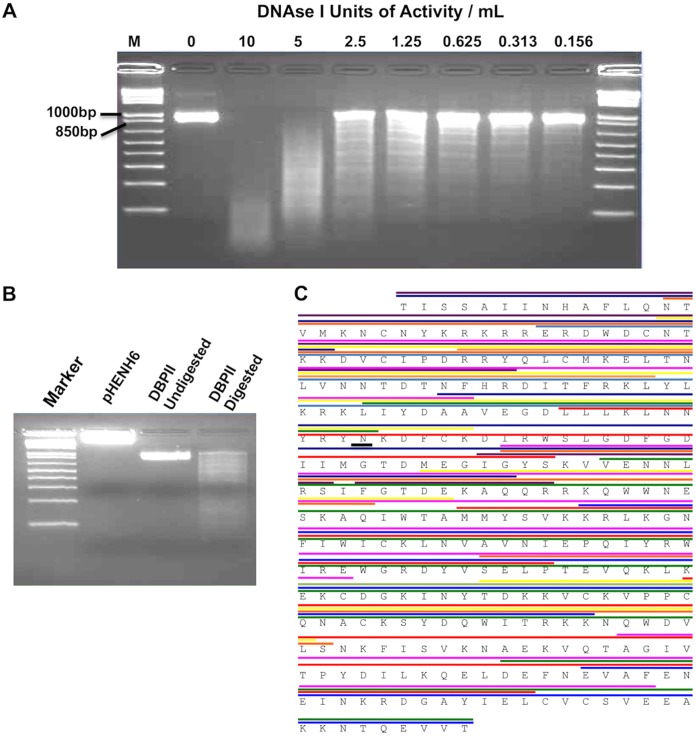
Generation of DBPII gene fragment library. DNA gels of DBPII Sal1 digested to make gene fragments. (A) The optimal DNase I concentration was evaluated to obtain the broadest spread of DBPII fragments. Lane M contains markers. (B) DBPII Sal1 fragments obtained by digestion with 5 U/ml of DNase I and blunt ended ligated into the pHENH6 phagemid vector and transformed into E. coli TG1. (C) Sequencing of PCR products generated by screening 40 individual clones revealed that the gene fragments in the library spanned the entire coding sequence, with no bias toward any particular region. The different colors represent the lengths of the various gene fragments identified.

Variable-length DBPII gene fragments were created by digestion of DNA encoding the complete open reading frame of DBPII with DNase I (Fig. 1). First, a pilot experiment was used to determine the best possible concentration of the enzyme to obtain the broadest spread of the DBPII fragments (Fig. 1A), and 5 U/ml was determined to be the optimal concentration (Fig. 1B). The digested fragments were cloned into the pHENH6 vector and successfully used to express a large diversity of random-length fragments of DBPII. The DBPII fragments were expressed on the surface of the engineered phagemid as chimeric proteins fused with a C-terminal c-*myc* epitope tag acting as an expression marker and the pIII minor coat protein. The recombinant phagemid library for DBPII was estimated to have 7 × 10^5^ PFU/μg of plasmid DNA gene fragments. Sequencing of 40 random clones from the library revealed that the fragments in the library spanned the entire coding sequence, and no bias toward any particular region was observed (Fig. 1C), although most of the fragments were ligated out of frame or in the incorrect orientation in accordance with random chance. Phage stocks generated from the gene fragment libraries were biopanned on the highly inhibitory MAb 3C9 and the poorly inhibitory MAb 3D10 to enrich for high-affinity binders to each of these antibodies.

### Biopanning of DBPII phage displayed fragment library for minimal epitopes reactive with anti-DBPII MAbs.

**FIG 2:**
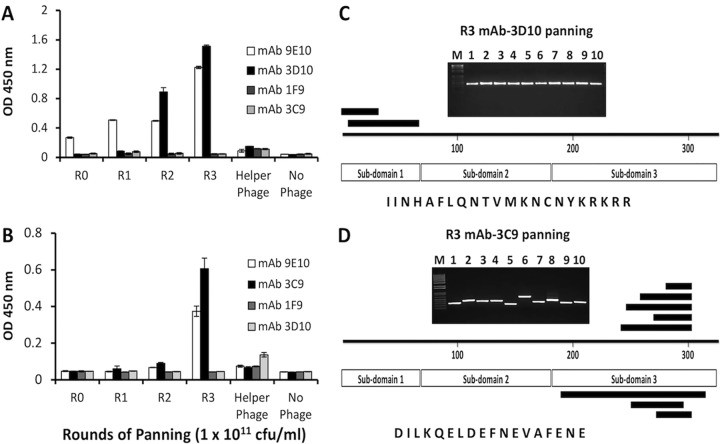
DBPII gene fragments identified through biopanning. (A and B) ELISA reactivity of phage clones enriched by successive panning on MAb 3D10 (A) and MAb 3C9 (B). A pool of phage from each round of panning was tested for binding to anti-DBPII MAbs 3D10 and 3C9 and the anti-c-*myc* epitope tag antibody MAb 9E10. The PfAMA-1-specific MAb 1F9 served as a negative-control antibody. The bars represent mean ODs of triplicate wells, and error bars indicate the standard deviations (SD). Individual clones (*n* = 10) from round 3 (R3) of panning on each of the MAbs were PCR amplified and sequenced. The positions of the various peptides identified are indicated in panels C and D for MAbs 3D10 and 3C9, respectively. The common consensus sequence identified from the phage clones by panning on each of the antibodies is shown.

Affinity selection of reactive phage to each target antibody was conducted through sequential rounds of panning by standard procedures. An antibody to the c-*myc* epitope tag, MAb 9E10, was used in ELISAs as a control to standardize library titer in each round of panning, and an anti-PfAMA1 antibody, MAb 1F9, served as a negative control (Fig. 2), while MAb 3D10 was used as a control to validate the phage display as a platform for epitope identification. Panning on MAb 3D10 selected for phage clones that bound well to MAb 3D10, but poorly to MAbs 3C9 and 1F9 (Fig. 2A). Inversely, panning on MAb 3C9 selected for phage clones that bound well to MAb 3C9 and poorly to MAbs 3D10 and 1F9 (Fig. 2B). Phage clones isolated from panning on all the antibodies bound well to MAb 9E10, indicating that the procedure had enriched for clones possessing DBPII coding sequence in frame and in the correct orientation.

Sequence analysis of 10 clones from each round of panning was used to identify the length and specificity of DBPII CDS inserts. Clones from the last rounds of panning tended to have inserts of similar sizes, while those of the early rounds tended to be more variable. Sequencing results further showed that all clones in the last round of panning (R3) were in frame and in the right orientation, corroborating ELISA results. Ten clones from the last rounds of panning on MAb 3D10 contained only two different sequences comprising fragments of subdomain 1. These overlapping fragments, which identified the minimal 3D10-reactive epitope as IINHAFLQNTVMKNCNYKRKRR represented surface residues on subdomain 1 of DBPII (Fig. 2C) and overlapped the previously identified minimal epitope determined by X-ray crystallography (45). From the panning on MAb 3C9, eight unique sequence fragments were obtained comprising overlapping fragments of subdomain 3, with DILKQELDEFNEVAFENE as the minimal reactive epitope (Fig. 2D). This epitope is highly conserved in P. vivax field isolates.

**FIG 3:**
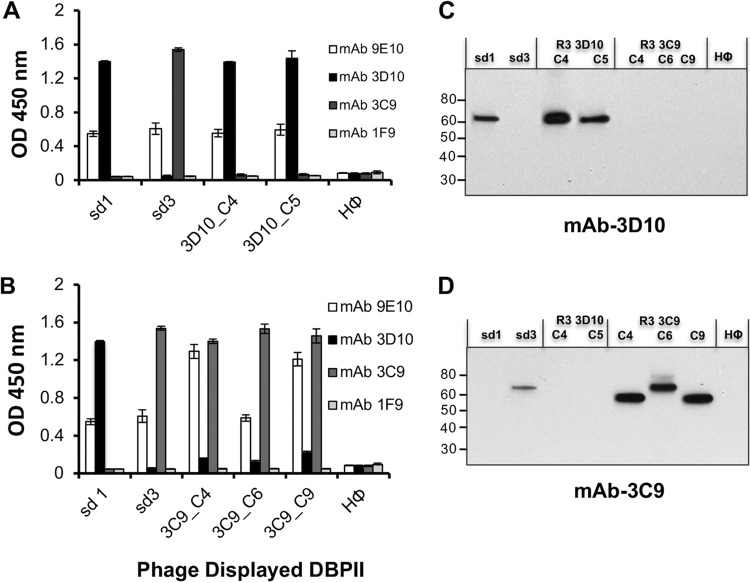
Cross reactivity of isolated phage clones with MAbs 3D10 and 3C9. (A to D) Two phage clones (clone 4 [C4] and C5) and three phage clones (C4, C6, and C9) from round 3 panning of the DBPII gene fragment library on MAbs 3D10 and 3C9, respectively, and phage clones expressing sd1 and sd3 fragments of DBPII were tested for cross-reactivity with the homologous and heterologous antibodies by ELISA (A and B) and immunoblot analysis (C and D). MAb 3D10 binds specifically to MAb 3D10-isolated phage clones and the sd1-expressing clones, while MAb 3C9 binds only to MAb 3C9-isolated clones and sd3-expressing clones. MAb 1F9 is a nonspecific anti-DBPII antibody used as a negative control, and MAb 9E10 is specific to the c-*myc* epitope of the phagemid. Each bar represents the mean OD_450_ for triplicate wells, and error bars represent SD.

Two and three phage clones from the DBPII gene fragment library enriched during round 3 panning on MAbs 3D10 and 3C9, respectively, were cultured, and each clone was tested for reactivity with the selecting MAb by ELISA and Western blotting (Fig. 3). Results from both assays indicated that phage clones isolated from panning on MAb 3D10 reacted specifically with the 3D10 MAb (Fig. 3A and C). Similarly, MAb 3C9 phage clones were found to react specifically with MAb 3C9 (Fig. 3B and D). The observed specificity suggested that the isolated phage clones displayed the minimal fragments of DBPII needed for the MAbs to bind their respective epitopes.

To further characterize the linear epitope for MAb 3D10, a random 20-mer peptide library termed Adlib1 (Adalta Pty Ltd.) was also used to pan on the antibody. The level of reactivity was assessed by ELISA after three rounds of panning. Rounds 1 to 3 reacted well with MAb 3D10, while its reactivity remained relatively poor to the negative isotype control, anti-AMA1 MAb 5G8 (see Fig. S1A in the supplemental material). Ten clones from round 3 of panning were sequenced, and three different sequences (mimotopes) were identified, each containing what may be a degenerate sequence motif (Fig. S1B). A recurrent motif within the 3D10 mimotopes was a hydrophilic three-residue motif YK(R/Y/E). Although the Adlib1 peptide library is unrelated to DBPII, the YKR motif matches a similar sequence motif within the 22-amino-acid sequence in subdomain 1 selected by MAb 3D10 on the DBPII gene fragment library (Fig. 2C).

10.1128/mSphere.00194-19.1 FIG S1 Panning a random peptide library on MAb 3D10. (A) ELISA showing reactivity of phage clones from rounds of panning on MAb 3D10. MAb 5G8 served as a negative-control antibody, while a MAb 5G8-positive binding phage clone served as a positive control (Pos C). The bars represent means for triplicate wells, while the error bars represent standard deviations (SD). (B) Alignment of a sequence of the MAb 3D10 binding epitope on DBPII sd1 from gene fragment library (top) and sequences of three mimotopes (M1, M2, and M3) from random peptide library with affinity for MAb 3D10. The residues in boldface type (underlined) show 3-amino-acid motifs common to the DBPII epitope and the mimotope sequences. Download FIG S1, TIF file, 0.3 MB. Copyright © 2019 George et al. 2019 George et al. This content is distributed under the terms of the Creative Commons Attribution 4.0 International license.

### Mutational analysis of the 3C9 epitope.

**FIG 4:**
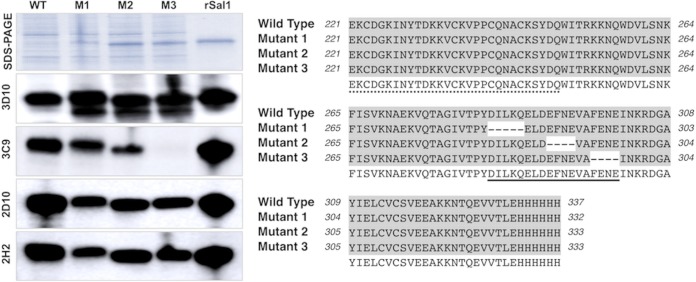
Binding of DBP monoclonal antibodies to DBPII-Sal1 mutants. (Left) Western blot analysis showing binding of MAbs 3D10, 3C9, 2D10, and 2H2 to Escherichia coli (BL21 star) expressing DBPII with mutations within the 3C9 epitope (left). Lanes: WT, wild type; M1, mutant 1; M2, mutant 2; M3, mutant 3; rSal1, purified rDBPII-Sal1. (Right) Sequences of a portion of subdomain 3 of the three mutants and the wild type are shown. The 3C9 epitope is underlined (solid line), 2D10 and 2H2 share an epitope (dotted line), and deleted residues within the 3C9 epitope are indicated by dashes.

To confirm the epitope identified through panning on MAb 3C9, DBPII-Sal1 with deleted residues at the beginning, middle, and end of the epitope were evaluated for binding to MAbs 3C9 and 3D10. Deletion of the DILKQ residues at the beginning (mutant 1) and EFNE residues in the middle (mutant 2) of the epitope, respectively, showed about 25% and 50% reduction in binding of MAb 3C9, while deletion of the FENE residues (mutant 3) at the end of the epitope completely inhibits MAb 3C9 binding (Fig. 4). On the other hand, MAbs 3D10 bound all three mutants and the wild type. Similarly, two other DBP inhibitory MAbs (2D10 and 2H2), located upstream of the 3C9 epitope also bound to all mutants and the wild type.

### Evaluation of immunogenicity of DBPII epitope peptides.

**FIG 5:**
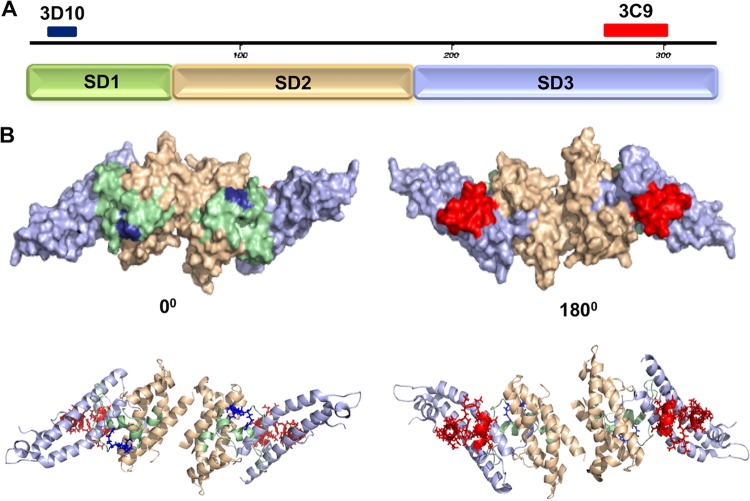
Putative epitopes of MAbs 3C9 and 3D10 mapped on 3D structure of DBPII. (A) The three subdomains (subdomain 1 [SD1] to SD3) are highlighted by color along with the putative epitopes of the inhibitory MAb 3C9 (red) on SD3 and the noninhibitory MAb 3D10 (blue) on SD1. (B) The 3D structure of DBPII is shown as the dimer required for DARC receptor recognition. The two views represent the front and back of the ligand for the surface model (top) and for secondary structure cartoon (bottom).

**FIG 6:**
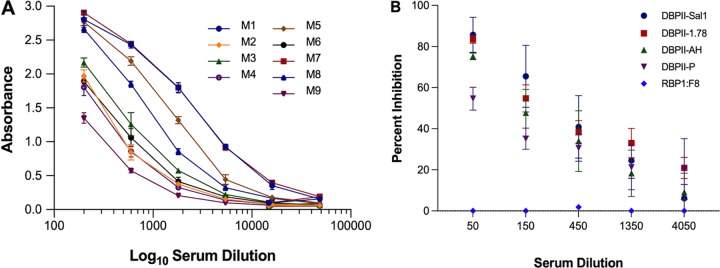
Immunogenicity of 3C9 epitope peptide. (A) Mice immunized with the peptide corresponding to the 3C9 epitope-generated antibodies reactive with rDBPII by ELISA. Each curve represents antibody response from individual mice. Each point on the curve represents the mean OD for triplicate wells, and error bars indicate standard deviations (SD). (B) Pooled mouse sera were tested by endpoint dilution for inhibition of DBPII-erythrocyte binding against allelic DBPII variants by standard *in vitro* COS7 assay. Each curve on the chart represents the means from two independent experiments with each dilution tested in triplicate. Error bars represent SD.

Both the 3D10 and 3C9 epitopes mapped to residues determined to be on the surface of the DBPII 3D crystal structure (Fig. 5). Since it appeared that the 3C9 epitope may retain its alpha helical secondary structure present in the native DBPII structure and 3D10 is at least partly a linear epitope, we sought to determine whether peptide immunogens of these minimal reactive epitopes could induce antibodies reactive with native DBPII. Particularly for the 3C9 epitope, we sought to determine whether the synthetic peptide of the epitope could retain sufficient native structure to elicit functional antibodies reactive to the native epitope and inhibitory against DBPII’s erythrocyte binding activity. The minimal peptides identified from panning with DBPII gene fragment library and the random peptide library were synthesized and conjugated to KLH carrier protein to immunize mice. ELISAs were used to determine reactivity of the antipeptide sera with the minimal peptide epitopes of MAbs 3D10 and 3C9. Serum from each mouse was tested for affinity to its homologous peptide. All peptides were immunogenic in mice and produced antibodies to the homologous peptides. Further, each antiserum was tested for affinity to rDBPII, and interestingly, mice immunized with 3C9 epitope peptide produced a measurable antibody response reactive with rDBPII (Table 1 and Fig. 6A).

**TABLE 1:** Epitopes identified through biopanning phage libraries on MAbs 3C9 and 3D10

Epitope	Peptide sequence^*a*^	ELISA data	
		Peptide	rDBPII Sal1
3C9	DILKQELDEFNEVAFENE	+	+
3D10	IINHAFLQNTVMKNCN**YKR**KRR	+	−
3D10-M1^*b*^	VGNLDFSRFHKSSLD**YKR**GQ	+	−
3D10-M2^*b*^	VKFTDR**YKY**SSMKGYARQGR	+	−
3D10-M3^*b*^	KINM**YKE**VRTRQLSVRPSPE	+	−
Control	TPDERYRELDSHAQNESC	+	−

a Degenerate sequence is highlighted in boldface type.

b Mimotopes identified by panning on random peptide library.

### Characterization of specificity and functional activity of anti-DBPII peptide serum antibodies.

To assess the anti-3C9 epitope peptide antibody activity, the peptide serum was evaluated for its potential to inhibit COS7 cell surface-expressed variant DBPII alleles (Sal1, P, 7.18, and AH) (see Table S1 in the supplemental material), from binding to DARC-positive erythrocytes. A concentration-dependent inhibition of DBPII-erythrocyte binding was observed (Fig. 6B).

10.1128/mSphere.00194-19.2 TABLE S1 DBPII variant alleles used in COS7 cell binding assay. Differences in amino acid residues between alleles compared to DBPII-Sal1 are indicated by letters, while similar residues are represented by periods. Download Table S1, PDF file, 0.1 MB. Copyright © 2019 George et al. 2019 George et al. This content is distributed under the terms of the Creative Commons Attribution 4.0 International license.

Statistical analysis using Dunn’s multiple-comparison analysis showed no significant differences in the overall inhibitory responses between the different alleles (*P* > 0.05). These results suggest that the 3C9 peptide produced a functional antibody response to this conserved region of DBPII within these alleles, although significant differences in inhibition were observed between the Sal1 and P alleles at high antibody concentrations (*P* = 0.03).

## DISCUSSION

DBP is vital for P. vivax merozoite invasion of Duffy-positive reticulocytes, making it a leading vaccine candidate against blood-stage vivax malaria (14). As the prime representative member of the DBL-EBP (18, 25), DBP has helped define the functional and immunological properties of this important family of malarial ligands (17, 27, 29, 32). One of the biologically important conundrums of DBP is that the conserved structure of the N-terminal cysteine-rich DBL ligand domain essential for receptor recognition is under immune selective pressure driving allelic variation that alters the antigenic character of epitope targets of protective antibodies (26, 56). This variation is mainly constrained to polymorphisms in the DBL domain that exhibits some variation by geographic region (57–59). Some polymorphic residues on DBPII are unique to a certain geographical region, while others are common among global vivax alleles, like residues K371E, D384G, E385K, K386N, N417K, L424I, W437R, and I503K (57, 58, 60, 61). Generally, variation occurs in nonessential residues flanking residues critical for receptor recognition, and variation in some residues is more important than in others (26, 35). Overall evidence indicates that variation plays an important role in strain-specific immunity to P. vivax DBP.

In previous studies, we determined that one of the primary dominant Bc epitopes reactive with functionally inhibitory human immune sera contains some of the most polymorphic residues (27, 30), and this epitope was subsequently determined to be a major determinant of strain-specific immunity in DBPII (37, 62, 63). In a recent study, we established that a synthetic DBPII-based vaccine termed DEKnull-2, which lacked the polymorphic residues in the ligand domain of DBPII was able to induce broadly binding-inhibitory anti-DBPII antibodies, capable of blocking merozoite invasion of reticulocytes (64). This suggests that targeting immune response to conserved functional epitopes within DBPII, which are targets of broadly BIAb is key to overcoming the challenge of strain-specific immunity associated with a DBP vaccine. Using X-ray crystallography, we identified the epitopes of three inhibitory anti-DBPII MAbs (2D10, 2H2, and 2C6) and a noninhibitory MAb (3D10) (44, 45). In this study, we used a different platform (phage display) to identify the epitope of a broadly inhibitory anti-DBP MAb 3C9 (44) for which a high-resolution epitope structure could not be elucidated. This platform is a great tool for elucidating the molecular nature of protein-protein interactions (46–48) and was previously used to successively map functional epitopes on PfAMA1, a leading candidate for asexual-stage P. falciparum malaria vaccine (48), and we have established the methodology for DBPII. Recombinant filamentous phage was engineered to display DBPII-Sal1 on its surface as part of the pIII capsid protein. Selective panning of recombinant phage libraries on antibodies was used to isolate target epitopes or peptide mimics. Positive epitope-containing clones reactive with the anti-DBPII antibodies were enriched by successive panning assays. Importantly, panning on MAb 3D10 identified an epitope previously identified and confirmed by X-ray crystallography (45), located in subdomain 1, thus validating this platform.

The specificity of the epitope identified for MAb 3C9, which is located in subdomain 3, was confirmed by mutational analysis (Fig. 4). Essential residues for MAb 3C9 binding were located toward the end of the epitope. Binding of MAbs 3D10, 2D10, and 2H2 to all three mutants validates the mutants and suggest that the mutations within the 3C9 epitope did not compromise the structure and conformation of the rest of the protein domains. It is important to note that MAbs 2D10 and 2H2, also located in subdomain 3 share an epitope, located about 35 residues upstream of the 3C9 epitope (45).

A surprising outcome from this study and our previous study mapping the epitopes of inhibitory anti-DBPII MAb (45) is the discovery that Bc epitope targets of highly inhibitory anti-DBP are located in subdomain 3 away from the dimer interface and residues determined to be critical for erythrocyte binding. The subdomain 3 epitope identified by the 3C9 MAb is independent of the epitopes identified by X-ray crystallography, further validating that this subdomain of the DBP ligand is sensitive to antibody functional inhibition and may serve as a key target for vaccine-elicited antibody.

Our previous analyses indicated that most inhibitory antibodies to DBPII are dependent on 3D conformation and reactivity as well as functional immunogenicity are lost when DBPII is denatured (40, 44, 64). However, the alpha helical nature of the 3C9 epitope suggested that it may retain some of its native conformation in a synthetic peptide. Therefore, to further evaluate its potential as a subunit synthetic vaccine, peptides representing the 3C9 and 3D10 epitopes were characterized by vaccination studies. The immunogenicity of the “protective” 3C9 epitope was compared to the minimal peptide epitope of 3D10. Although 3D10 represented the highest titer MAb within the anti-DBP panel created, and reacted equally to all DBP alleles tested by ELISA, it has virtually no ability to block DBPII-erythrocyte binding (44).

Various studies have examined the functional efficacy of vaccine-induced anti-DBP antibodies to block DBP-erythrocyte binding or inhibition of merozoite invasion of erythrocytes (62, 65–67). As a general rule, properly refolded rDBPII capable of erythrocyte binding activity is required for induction of inhibitory anti-DBPII antibodies. Indeed, the epitope targets of the protective MAb 3C9 (conformational) and nonprotective MAb 3D10 (partially linear) are consistent with this observation. In our approach, the DBP peptide immunogens, consisting of the synthetic linear peptides of the 3C9 or 3D10 epitopes conjugated to carrier KLH, were used to immunize mice. The 3D10 immunogens included mimotope peptides of epitope targets isolated from a random peptide library. Surprisingly, we found that a 3C9 epitope peptide successfully induced an antibody response reactive to the immunizing peptide, the refolded protein, as well as inhibitory to DBPII-erythrocyte binding. This result suggests that the 3C9 linear peptide has some inherent structural tendency to assume its 3D conformation displayed on the surface of native DBP and is the target of inhibitory antibody. Although this epitope is conserved among currently available sequences from clinical isolates, in our original characterization of MAb 3C9 (44), its reactivity to different DBP allelic variants tested showed some degree of variability, indicating that the antibody’s specificity may be altered by conformational changes due to variation outside the targeted epitope.

Structure-based immunogen design for malaria vaccine development (structural vaccinology) using specific functional epitopes of antigens has been attracting much attention recently (68). The down side of this approach is the low immunogenicity of the peptide epitopes used. Generally, both B-cell and T-cell responses play a role in naturally acquired immunity to malaria. Thus, the immunogenicity and functional activity of epitopes such as the 3C9 epitope can be enhanced (i) by incorporating a universal T-helper epitope (such as the P2 tetanus toxoid-derived T-cell epitope) (69), (ii) by using adjuvants such as TLR agonists (TLR4 and TLR5) (70) for immunization, which have been successfully used to enhance immunogenicity of a B-cell epitope of PvMSP9 and the prophylactic L2-based HPV vaccine, respectively, or (iii) by packaging the epitope on viruses and virus-like particles (VLPs) by presenting the peptide as an immunogen at high densities on the surface of the viral particle which elicit stronger, and longer-lasting immune responses. This platform has been used as the basis for developing the human papillomavirus (HPV) vaccine (71).

Thus, conserved epitopes like the 3C9 epitope and other previously identified epitopes capable of eliciting protective antibodies (45), provide critical motifs that should be retained in a DBP-based subunit vaccine design against asexual-stage vivax malaria. This can be achieved by creating an epitope scaffold immunogen with flexible backbones (multiepitope peptide vaccine) through conjugation of the peptide epitopes in a single construct (72–74). Such a vaccine will elicit inhibitory antibodies specific for each conserved epitope and reduce immune evasion mechanisms associated with genetic variants in the DBPII gene.

## MATERIALS AND METHODS

### Generation of phage-displayed DBPII gene random fragment library.

The full-length PCR product (20 μg) from DBPII Sal1 haplotype was digested with DNase I at 5 U/ml. The resulting fragments were purified by sodium acetate precipitation, and ragged ends were blunted with a Vent polymerase (NEB). The pHENH6 vector was digested with PstI (NEB) and also blunted with Vent polymerase. The resulting products from the randomly fragmented DBPII were cloned into the treated pHENH6 vector and then into Escherichiacoli TG1 by electroporation. The distribution of the fragments in the library was determined by PCR and sequencing of 40 random clones. The library was then grown in 20 ml of 2× Tryptone-yeast extract broth containing 50 μg/ml of ampicillin at 37°C with shaking until an optical density at 600 nm (OD_600_) of 0.6 was attained. M13K07 helper phage (1 × 10^11^ PFU) was added and cultured for an extra 1 h at 37°C without shaking to allow infection. The entire culture was then transferred into 400 ml of super broth containing 70 μg/ml kanamycin and 50 μg/ml ampicillin and incubated at 37°C with shaking overnight. The bacteria were pelleted by centrifugation at 8,000 × *g* for 15 min, and the phage was precipitated from the supernatant with PEG-NaCl as described previously (75, 76). The phage was suspended in 1 ml of PBS and used subsequently or stored at −80°C.

### Panning of phage-displayed DBPII gene fragment library.

Affinity panning of DBPII Sal1 gene fragment to anti-DBPII MAbs 3D10 and 3C9 was conducted as described previously (48). Briefly, 10 wells of Maxisorp-Nunc ELISA plate were coated overnight at 4°C with 100 μl per well of test MAb at a final concentration of 2.5 μg/ml diluted in PBS. The plates were washed twice with PBS, and unbound surfaces were blocked with 200 μl of 5% skim milk in PBS for 2 h at room temperature. After another round of washing, approximately 1 × 10^11^ phage/well in 1% skim milk in PBS were added to wells and incubated for 1 h at room temperature. Nonadherent phage particles were washed off twice with PBS/0.05% Tween 20. The bound phage was eluted with 100 μl of 0.1 M glycine (pH 2.2) and immediately added to 10 ml of log-phase E. coli TG1 cells and cultured for 30 min at 37°C for infection to occur. Ampicillin was added to 50 μg/ml, and the culture was incubated for another 30 min at 37°C before addition of the M13 helper phage and incubated for another 1 h at 37°C. The culture was then transferred to 200 ml super broth containing 50 μg/ml of ampicillin and 70 μg/ml of kanamycin and incubated at 37°C overnight with shaking at 200 rpm. The phage was harvested by centrifugation at 8,000 × *g* for 15 min, and another round of panning was conducted for a total of up to four panning cycles per antibody. Ten clones from each round of panning were selected, and their inserts were amplified by PCR and sequenced to determine the identity of the fragments of DBPII that bind to each MAb.

### Panning a random peptide library on MAb 3D10.

Panning on MAb 3D10 was performed using a random 20-mer peptide library (courtesy of M. Foley and R. F. Anders, La Trobe University, Australia; Adalta Pty Ltd.) as described previously (48, 75). Briefly, phage peptide library at 10^11^ phage/well was incubated with MAb 3D10-coated wells for 1 h at room temperature. Eluted phage was allowed to reinfect E. coli K91 grown to log phase in 10 ml Tryptone-yeast extract medium at 37°C for 1 h and then grown overnight in 200 ml of super broth with 40 μg/ml of tetracycline. Phage was harvested, and further rounds of panning were conducted as described above. Clones isolated from round 3 were sequenced to determine the peptide sequences.

### ELISA of phage displayed rounds of panning.

Enrichment ELISA was performed as described previously (48). Briefly, 100 μl of anti-DBP MAbs at 2.5 μg/ml in PBS was coated onto the wells of 96-well microtiter plates (Maxisorp-Nunc) overnight at 4°C. Anti-PfAMA1 specific MAb 1F9 or 5G8 (48) was coated on each plate as a negative-control antibody. The plates were washed twice with PBS 0.05% Tween 20 and blocked for 2 h at room temperature with 200 μl/well of 10% skim milk diluted in wash buffer. After three washes, phage diluted in PBS/1% milk to 1 × 10^11^ PFU/ml was added in triplicates and incubated for 2 h at room temperature. The plates were washed four times, and the wells were incubated with HRP-conjugated anti-M13 MAb (GE Life Sciences) for 1 h at room temperature. The plates were then washed five times, and bound phage was detected by incubating wells with 100 μl of H_2_O_2-_activated TMB substrate (Sigma). The reaction was stopped with 100 μl of 2 N H_2_SO_4_, and the plates were read at 450-nm absorbance.

### Immunoblots.

Approximately 1 × 10^12^ phage particles were boiled in SDS-PAGE sample buffer for 3 min, separated by SDS-PAGE, and electrophoretically transferred onto a nitrocellulose membrane. The membrane was blocked overnight in 10% skim milk diluted in PBS/Tween 20 and probed with anti-DBPII MAbs. The membrane was then washed with PBS/Tween 20 before incubating with an HRP-conjugated anti-mouse secondary antibody. The bound antibody was detected by enhanced chemiluminescence (ECL) substrate (Amersham).

### Mutational analysis.

With the DBPII-Sal1 strain as the template for mutagenesis, three different mutants were created within the 3C9 epitope by deleting the DILKQ, EFNE, and FENE residues at the beginning, middle, and end of the epitope, respectively, using the Q5 site-directed mutagenesis kit (NEB) according to the manufacturer’s protocol. Mutations were confirmed by sequencing. The mutants expressed in Escherichia coli BL21 Star (NEB) were screened by Western blotting for reactivity with MAbs 3C9, 3D10, and two other DBP inhibitory antibodies (MAbs 2D10 and 2H2), previously shown by cocrystallization studies to share an epitope (45).

### Immunizations.

Peptides corresponding to sequences of the epitopes identified from panning on the gene fragment library (3C9, from DBPII subdomain 3; 3D10, from DBPII subdomain 1) and mimotopes from panning on the random peptide (3D10-M1, 3D10-M2, and 3D10-M3) (Table 1) were commercially synthesized (Pacific Immunology) and conjugated to keyhole limpet hemocyanin (KLH) using maleimide-activated mcKLH (ThermoScientific), following the manufacturer’s specifications. The conjugated peptides were used to immunize 6- to 8-week-old BALB/c mice (Harlan) to raise immune sera. All animals were handled in compliance with good animal practice, and all experimental procedures were performed in accordance with IACUC protocols approved by the Division of Research Integrity and Compliance, University of South Florida. Briefly, mice in all groups (*n* = 10) were bled for preimmune sera, and each mouse was immunized three times at 3-week intervals with 5 μg of KLH-conjugated peptide emulsified in Titermax Gold adjuvant. Each animal received a 50-μl antigen-adjuvant mix administered subcutaneously at the base of the tail. Mice immunized with KLH and adjuvant alone served as control. All mice were bled for final serum 4 weeks after the second boost.

### Measurement of antibody titers.

Total anti-peptide IgG titers in the sera of each group was evaluated by endpoint titration ELISA against recombinant DBPII Sal1 and homologous peptide conjugated to bovine serum albumin (BSA). Imject Maleimide-Activated BSA Spin kit (ThermoScientific) was used as specified by the manufacturer for peptide conjugation. ELISAs were conducted as described previously (44), 0.1 μg of peptide conjugated to BSA or 0.25 μg of recombinant antigen was used to coat each well of a microtiter plate overnight and blocked with 5% skim milk PBS/0.05% Tween 20 detected by threefold dilution of mouse sera was used starting at 1:200. Bound antibodies were detected using alkaline phosphatase-conjugated anti-mouse antibody (Kirkegaard & Perry Laboratories). Preimmune serum, at the lowest dilution, served as background and was subtracted.

### Measurement of functional inhibition of DBP-erythrocyte binding.

Pooled immune serum from mice that recognized rDBPII Sal1 by ELISA was tested further for inhibition of DBPII-erythrocyte binding by a modified version of the standard *in vitro* COS7 cell assay (29, 40). An expression plasmid, pEGFP-N1 (where EGFP is enhanced green fluorescent protein) (Clontech), was used to target variant DBPII alleles (Sal1, P, 7.18, and AH) (44, 62) (see Table S1 in the supplemental material) onto the surfaces of transfected COS7 cells as fusion proteins to the N terminus of EGFP. The expressed protein mimics the native protein on the surface of the parasite. Details of the assay and the different DBPII alleles were previously reported (62). COS7 cells transfected with plasmid expressing reticulocyte binding protein 1 (RBP1) and an empty plasmid were used as controls. Rosettes (COS7 cells with adherent erythrocytes) were determined by microscopy as positive when adherent erythrocytes covered at least 50% of the cell surface. The ability of the serum to inhibit binding of erythrocytes to DBPII expressed on the surfaces of transfected COS7 cells was determined by incubating transfected COS7 cells with triple fold dilutions of the serum prior to incubation with DARC-positive human erythrocytes. Binding inhibition was scored as the percentage of rosettes in wells of transfected COS7 cells in the presence of immune serum relative to wells incubated with preimmune serum (62). Differences in the inhibitory responses of the immune serum to the different alleles was compared by one-way analysis of variance (ANOVA) and multiple-comparison analysis by Tukey’s test using SAS software.
