# Supplementary figures and images for: Identification of an Immunogenic Broadly Inhibitory Surface Epitope of the Plasmodium vivax Duffy Binding Protein Ligand Domain
Source: mSphere. 2019 May 15;4(3):e00194-19. doi: 10.1128/mSphere.00194-19 (PMC6520440; doi:10.1128/mSphere.00194-19)

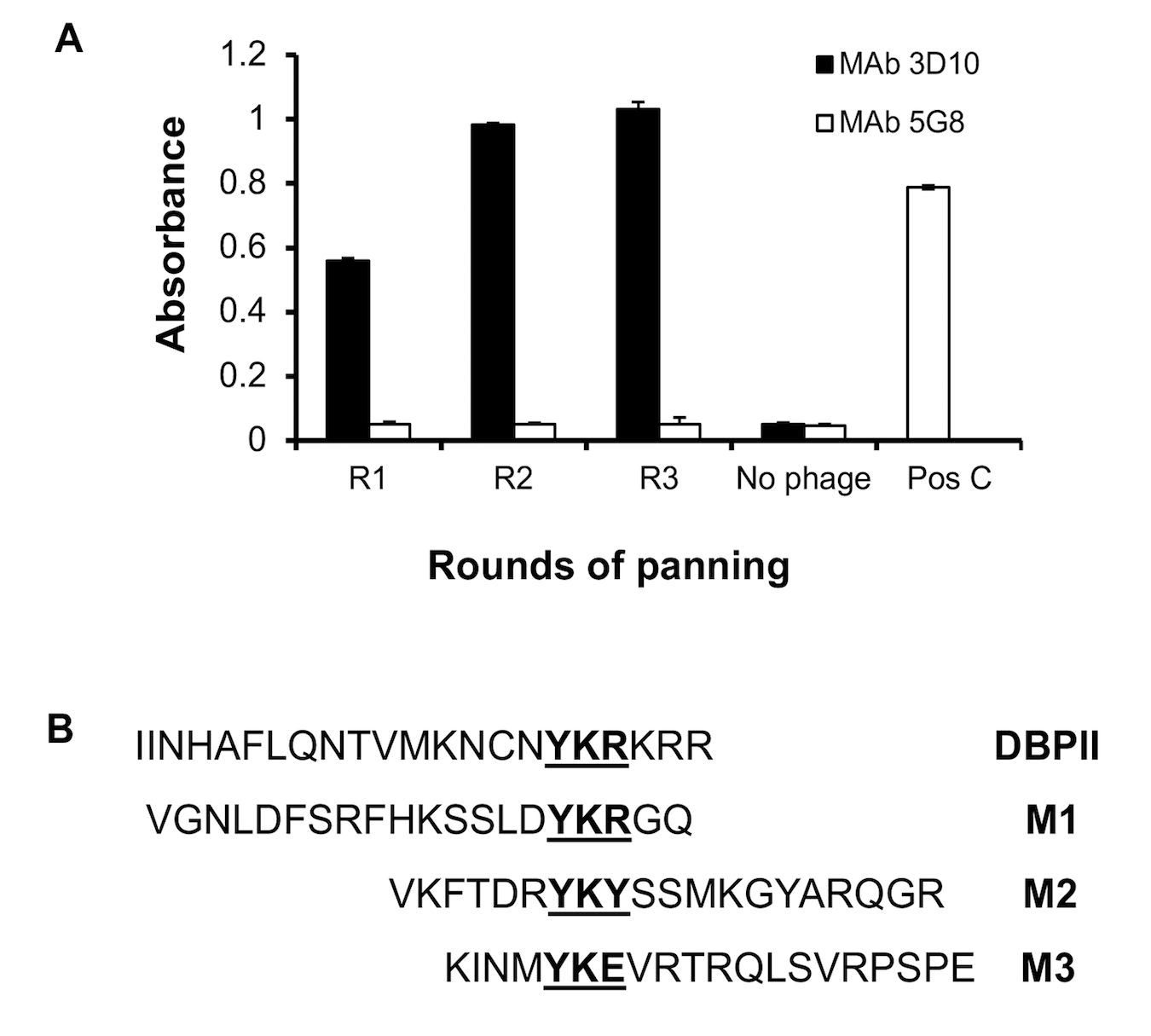

Supplement: FIG S1 [file mSphere.00194-19-sf001.tif]
