# Supplementary material for: Identification of an Immunogenic Broadly Inhibitory Surface Epitope of the Plasmodium vivax Duffy Binding Protein Ligand Domain
Source: mSphere. 2019 May 15;4(3):e00194-19. doi: 10.1128/mSphere.00194-19 (PMC6520440; doi:10.1128/mSphere.00194-19)
Supplement: TABLE S1 [file mSphere.00194-19-st001.pdf]

DBPII Allele	Accession #	Residue Position																						
		308	319	333	369	371	375	384	385	386	387	388	389	390	391	392	393	417	424	437	447	454	992	503	505
DBPII-Sal 1	P22290.2	R	R	L	Y	K	N	D	E	K	A	Q	Q	R	R	K	Q	N	L	W	S	Q	K	I	V
DBPII-7.18	AAL79051.1	S	.	.	.	.	.	G	.	Q	.	.	.	.	.	.	.	K	I	R	.	.	.	K	.
DBPII-AH	AAY34130.1	S	.		.	E	.	G	.	Q	.	.	.	.	.	.	.	K	I	R	.	.	.	K	.
DBPII-P	AAL79073.1	S	.	F	.	.	D	G	K	N	.	.	.	H	.	.	.	K	I	.	.	.	.	K	.
